# Quantitative CT radiomics-based models for prediction of haematoma expansion and poor functional outcome in primary intracerebral haemorrhage

**DOI:** 10.1007/s00330-021-07826-9

**Published:** 2021-04-16

**Authors:** Stefan Pszczolkowski, José P. Manzano-Patrón, Zhe K. Law, Kailash Krishnan, Azlinawati Ali, Philip M. Bath, Nikola Sprigg, Rob A. Dineen

**Affiliations:** 1 Radiological Sciences, Division of Clinical Neuroscience, Precision Imaging Beacon, University of Nottingham, Queen’s Medical Centre, Derby Road, Nottingham, NG7 2UH UK; 2 Stroke Trials Unit, Division of Clinical Neuroscience, University of Nottingham, Nottingham, UK; 3 Department of Medicine, National University of Malaysia, Kuala Lumpur, Malaysia; 4 Stroke, Nottingham University Hospitals NHS Trust, Nottingham, UK; 5 Sir Peter Mansfield Imaging Centre, University of Nottingham, Nottingham, UK; 6 NIHR Nottingham Biomedical Research Centre, Nottingham, UK

**Keywords:** Radiomics, Cerebral parenchymal hemorrhage, Linear models, Predictive medicine

## Abstract

**Objectives:**

To test radiomics-based features extracted from noncontrast CT of patients with spontaneous intracerebral haemorrhage for prediction of haematoma expansion and poor functional outcome and compare them with radiological signs and clinical factors.

**Materials and methods:**

Seven hundred fifty-four radiomics-based features were extracted from 1732 scans derived from the TICH-2 multicentre clinical trial. Features were harmonised and a correlation-based feature selection was applied. Different elastic-net parameterisations were tested to assess the predictive performance of the selected radiomics-based features using grid optimisation. For comparison, the same procedure was run using radiological signs and clinical factors separately. Models trained with radiomics-based features combined with radiological signs or clinical factors were tested. Predictive performance was evaluated using the area under the receiver operating characteristic curve (AUC) score.

**Results:**

The optimal radiomics-based model showed an AUC of 0.693 for haematoma expansion and an AUC of 0.783 for poor functional outcome. Models with radiological signs alone yielded substantial reductions in sensitivity. Combining radiomics-based features and radiological signs did not provide any improvement over radiomics-based features alone. Models with clinical factors had similar performance compared to using radiomics-based features, albeit with low sensitivity for haematoma expansion. Performance of radiomics-based features was boosted by incorporating clinical factors, with time from onset to scan and age being the most important contributors for haematoma expansion and poor functional outcome prediction, respectively.

**Conclusion:**

Radiomics-based features perform better than radiological signs and similarly to clinical factors on the prediction of haematoma expansion and poor functional outcome. Moreover, combining radiomics-based features with clinical factors improves their performance.

**Key Points:**

*• Linear models based on CT radiomics-based features perform better than radiological signs on the prediction of haematoma expansion and poor functional outcome in the context of intracerebral haemorrhage.*

*• Linear models based on CT radiomics-based features perform similarly to clinical factors known to be good predictors. However, combining these clinical factors with radiomics-based features increases their predictive performance.*

**Supplementary Information:**

The online version contains supplementary material available at 10.1007/s00330-021-07826-9.

## Introduction

Haematoma expansion (HE) is a complication that affects around one in five people with spontaneous intracerebral haemorrhage (ICH) during the first 24 h after symptom onset [1]. Additionally, HE is associated with poor functional outcome and is a therapeutic target for improving outcome [2, 3]. Identifying those with a high risk of HE may allow selective targeting of patients with high chance of benefit in clinical trials testing haemostatic therapies.

Computed tomography angiography (CTA) spot sign has been validated as good predictor of HE in ICH [4–6]. However, noncontrast computed tomography (NCCT) is still the standard of care for acute stroke in most care settings worldwide; thus, CTA is not routinely performed. NCCT signs such as blend sign [7], black hole sign [8], hypodensities [9, 10], island sign [11], and swirl sign [12] have been proposed as alternative predictors of haematoma expansion and/or poor functional outcome. Nonetheless, those predictors suffer from intra- and interobserver variability, have variable definitions, and present modest sensitivity. For example, Law et al [13] reported sensitivities of 11.4–39.5% for haematoma expansion and 14.3–39.2% for poor functional outcome. These limitations highlight the need for alternative quantitative approaches that are performed automatically and may show better predictive performance.

A recent meta-analysis found that time from symptom onset to baseline CT imaging, baseline intracerebral haemorrhage volume, antiplatelet use, and anticoagulant use were important factors for the prediction of HE in the context of primary ICH [14]. Despite their importance, these clinical factors may not cover the full spectrum of predictive information that can be obtained from patients. Therefore, providing an approach that can automatically extract features from images may provide valuable complementary information to aid in the prediction.

Radiomics is a relatively recent quantitative approach in which a large number of features, such as intensity statistics, shape descriptors, or texture measurements, are extracted from radiological images to then be tested as predictors of outcomes [15, 16]. We hypothesise that radiomics-based features predict haematoma expansion and poor functional outcome, since they may capture not only characteristics of the haematoma known to relate to instability (such as heterogeneity or complex shape) but also subtle characteristics not readily appreciable to the naked eye. Recent studies have applied a radiomics-based approach to prediction of HE [17–19]; however, these studies were relatively small (the largest included just over 250 subjects) and are based on either a single centre [17, 19] or just 4 centres [18]. While useful to demonstrate the feasibility of the approach, the generalisability of the results is unclear. In this paper, we investigate the use of NCCT radiomics-based features and generalised linear models for prediction of both HE and poor functional outcome, in a retrospective analysis of data acquired prospectively in a large international multicentre randomised controlled trial in ICH. We also explore the predictive relation of our radiomics-based model with both radiological signs and clinical factors.

## Materials and methods

### Intracerebral haemorrhage subjects

**Fig. 1:**
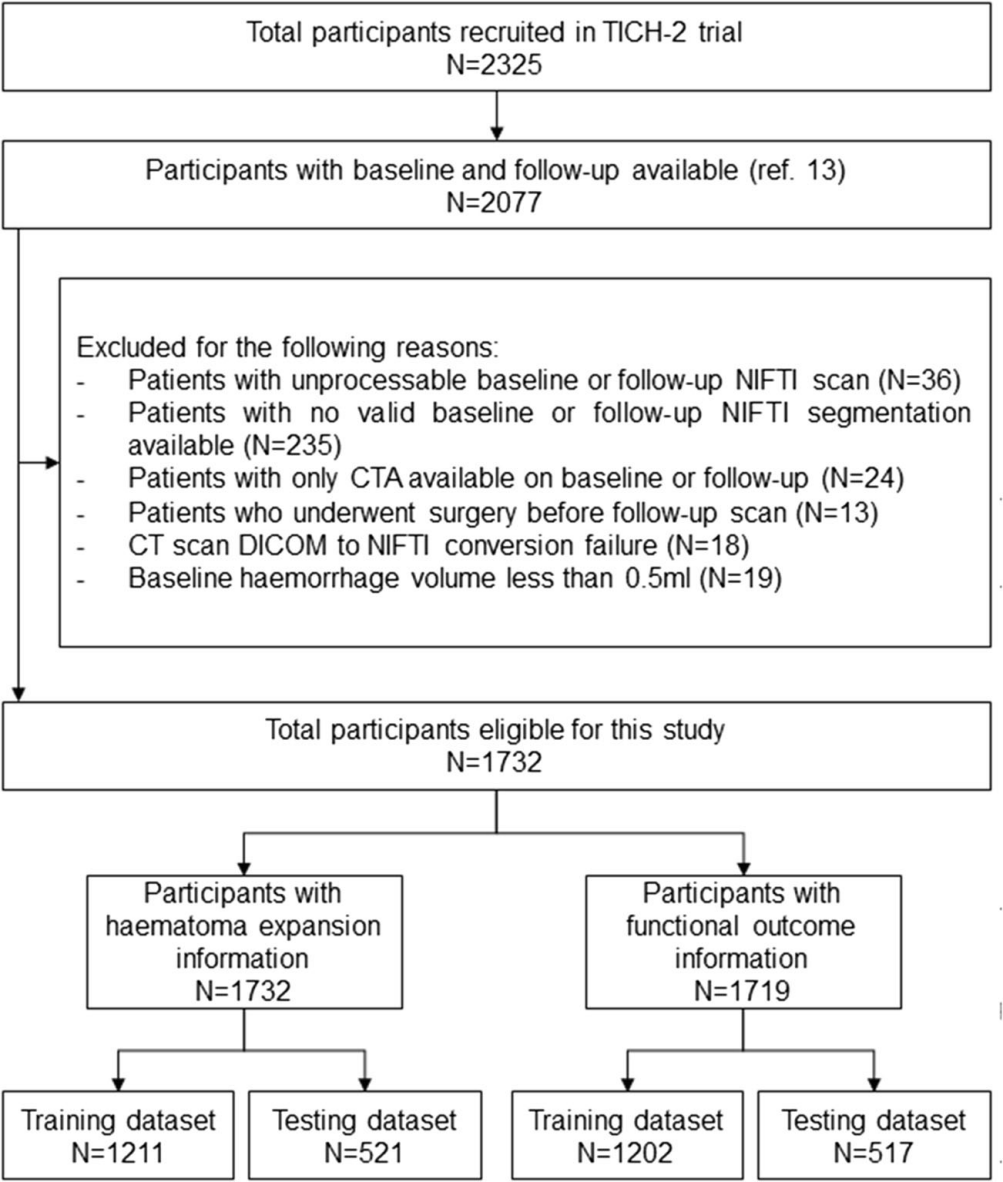
Study inclusion flowchart

We retrospectively included participants recruited prospectively to the TICH-2 international randomised, placebo-controlled clinical trial (ISRCTN93732214) [20]. This trial tested the efficacy and safety of intravenous tranexamic acid in people with acute spontaneous intracerebral haemorrhage presenting within 8 h of symptom onset. Primary outcome was functional status at day 90 measured by modified Rankin scale. Ethical approval for TICH-2 was obtained from the local institutional review board and informed consent was obtained before enrolment, either from the participant or one of their relatives. The rationale, protocol, and inclusion/exclusion criteria for the TICH-2 trial have been reported elsewhere [21]. All 2077 TICH-2 trial participants that had valid baseline and follow-up scans and have been previously reported [13] were eligible for inclusion in this analysis. We excluded 345 participants for our analysis due to clinical and technical reasons (Fig. 1), yielding a total of 1732 participants. Finally, all analyses were performed on a stratified semi-random split of the participants into a training set (*N* = 1211, 70%) and testing set (*N* = 521, 30%), forcing both sets to be age- and gender-matched.

### Image acquisition

Noncontrast CT (NCCT) brain scans were acquired as part of routine clinical care at each of the 124 centres participating in TICH-2 following their local protocol. Baseline scans were acquired before randomisation and follow-up scans were acquired after 24 ± 12 h [21]. There were no restrictions on scanner manufacturer, scanner settings, or slice thickness. Nevertheless, only axial scans were accepted.

### Feature extraction

The proposed feature extraction process follows the image processing guidelines of the image biomarker standardisation initiative (IBSI) [22, 23]. Firstly, semi-automated volumetric segmentation of intracerebral haemorrhage, perihaematomal oedema, and intraventricular haemorrhage was performed from each baseline and follow-up NCCT scans by one of three independent experienced stroke imaging researchers (Z.K.L., K.K., and A.A.), who were blinded to clinical data, using ITK-SNAP version 3.6.0 (http://www.itksnap.org), with manual editing as required. The raters also classified each baseline NCCT scan as positive or negative for the presence of radiological markers (blend sign [7], black hole sign [8], hypodensities [9, 10], and island signs [11]). Reliability assessments for the haematoma volumetric measurement and radiological marker interpretation for these raters have been published previously [13].

Secondly, images were resampled to 1-mm isotropic voxel size and additional filtered versions were computed using Laplacian of Gaussian and Wavelet filters (see Supplementary Materials). A total of 754 NCCT radiomics-based features were subsequently extracted from the original and filtered scans (see Supplementary Table S1) using MATLAB R2019b. Seven hundred fifty-two of them were taken from the area defined as intracerebral haemorrhage on the baseline scan, using a third-party package (https://github.com/mvallieres/radiomics) [24] for textural features, and in-house code for first-order and shape-based features (see Supplementary Materials for code snippets). The remaining two features correspond to baseline perihaematomal oedema volume (mL) and baseline intraventricular haemorrhage volume (mL). Additionally, we incorporated the TICH-2 treatment allocation (either tranexamic acid or placebo) to the radiomics features as a covariate of no interest.

### Feature processing

Each feature vector was harmonised using the MATLAB version of the ComBat harmonisation package (https://github.com/Jfortin1/ComBatHarmonization) [25–27] with parametric adjustments to remove possible batch effects of slice thickness in the radiomics computation. This method has already been tested before for harmonisation of NCCT radiomics [28]. We utilised three batches: (1) slice thickness < 2 mm; (2) slice thickness ≥ 2 mm and < 4 mm; and (3) slice thickness ≥ 4mm. Additionally, age and gender were utilised as biological covariates. Harmonisation was performed first on the training set and the same parameters were then applied to harmonise the testing set.

**Fig. 2:**
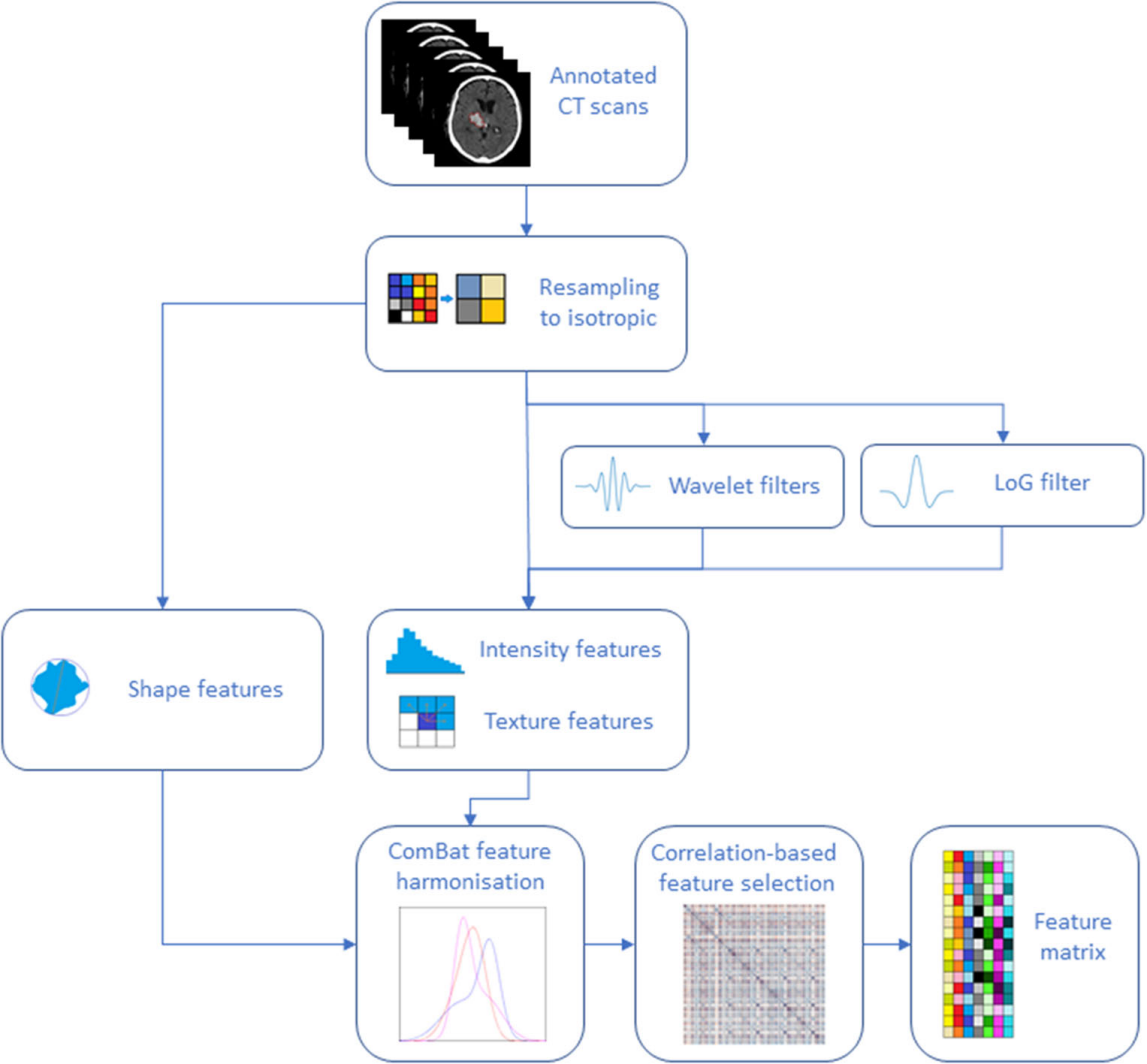
Feature extraction process flowchart. NCCT scans and their annotations are resampled to 1mm isotropic. Shape features are extracted from the resampled annotations and intensity and texture features are extracted from the resampled original and filtered images. This set of features, together with ultra-early haematoma growth are harmonised and the final set of uncorrelated features is then computed using a correlation-based filtering method. NCCT, noncontrast computed tomography; LoG, Laplacian of Gaussian

Also, an iterative feature selection procedure was run in which; for every pair of variables with absolute correlation > 0.9, the one with the largest mean absolute correlation is removed. After removing each variable, the average correlations were recomputed for the next iteration. This procedure was performed on the training data only and reduced the feature set to 218 unrelated features (see Supplementary Table S2). Figure 2 depicts the feature extraction process.

### Generalised linear model construction

**Fig. 3:**
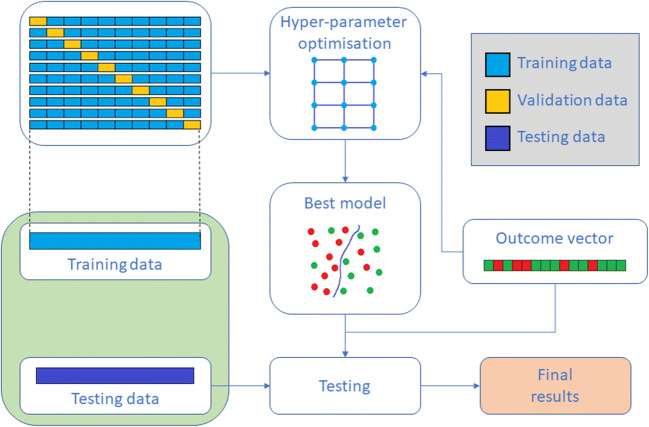
Training and testing procedure. The training UK data is split into 10 non-overlapping folds and 10 different models are trained for each value of the hyperparameter α, using each fold as validation data once. The model that shows the greatest AUC is selected for testing using the non-UK holdout data

We computed a generalised linear model (GLM) via elastic-net regularisation [29] with standardisation and log-loss score as energy function. To this end, we performed an exhaustive search-grid optimisation procedure with stratified 10-fold cross-validation (Fig. 3) using the H2O platform v3.26.0.2 (www.h2o.ai) and R software v3.6.3 (www.r-project.org). This search was carried out over the *α* blending hyperparameter of elastic-net regularisation, with values ranging from 0 (Ridge regression) to 1 (LASSO regression) in increments of 0.1 (see Supplementary Materials). Outcomes of interest were haematoma expansion, defined as volumetric growth of > 6 mL or > 33% on the follow-up scan, and poor functional outcome defined as modified Rankin scale of 4 to 6 at day 90 [20]. Finally, the optimal GLMs were chosen based on their cross-validation area under the receiver operating characteristic curve (AUC) score.

For comparison purposes, the same hyperparameter optimisation was performed using the presence of radiological signs alone as binary feature set and also using demographic and clinical information previously found to be predictive [14, 30]. The radiological markers used were the blend sign, black hole sign, hypodensities, and island signs. Demographic and clinical factors were age (years), gender (male/female), time from onset to baseline scan (hours), baseline haematoma volume (mL), antiplatelet use (yes/no), and ultra-early haematoma growth (baseline haemorrhage volume over time from onset to baseline scan). Anticoagulant use was also found to be predictive by Al-Shahi Salman et al [14]; however, this was an exclusion criterion of TICH-2 and hence not included. We also carried out the analysis using radiomics-based features combined with radiological signs and with clinical factors independently.

We also assessed our study using the radiomics quality score [31] (See Supplementary Materials for details). The score was 13 (36.11%), which is higher than the median score obtained on a recent systematic review of 51 cancer studies [32]. One of the main criteria affecting our score was the fact that despite TICH-2 being a prospective trial, it was not initially devised with radiomics in mind.

## Results

### Participant characteristics analysis

Participant demographics are summarised in Table 1 for haematoma expansion and Table 2 for poor functional outcome. Of the 1732 participants, 13 had no functional outcome information available and were excluded from the corresponding analysis. This accounts for the difference in the number of subjects between Tables 1 and 2. There was no significant difference in age, gender, or any of the included variables between training and testing sets (all *p* > .05). Finally, no statistical difference (all *p* > .05) in treatment allocation proportions was found between both sets.

**Table 1:** Patient characteristics for the training and testing datasets with respect to haematoma expansion. Data are number (%), mean (SD), or median (IQR). ^‡^*p* value between testing and training datasets

	Testing dataset (*N* = 521)			Training dataset (*N* = 1211)			
	Haematoma expansion	No haematoma expansion	*p* value	Haematoma expansion	No haematoma expansion	*p* value	*p* value^‡^
Number	137 (26.3%)	384 (73.7%)		337 (27.8%)	874 (72.2%)		
Age, years	70.03 (13.13)	68.52 (12.95)	.244	69.94 (14.26)	68.39 (13.72)	.082	.894
Gender, male	82 (59.9%)	211 (54.9%)	.367	200 (59.3%)	481 (55.0%)	.196	.999
Treatment allocation, placebo	77 (56.2%)	185 (48.2%)	.112	183 (54.3%)	424 (48.5%)	.073	.950
Baseline volume, mL	20.88 (7.73–40.00)	10.09 (5.12–20.65)	< .001	20.84 (7.50–42.98)	10.15 (4.68–20.52)	< .001	.851
Onset to CT scan, h	1.73 (1.30–2.51)	2.03 (1.40–3.13)	.004	1.80 (1.27– 2.47)	2.00 (1.38–3.00)	< .001	.564
Ultra-early haematoma growth, mL/h	11.06 (4.74–22.37)	5.12 (2.25–10.77)	< .001	10.81 (4.32–23.40)	5.04 (2.10–11.68)	< .001	.887
Systolic blood pressure, mm Hg	172.01 (30.32)	174.25 (28.78)	.440	173.73 (28.58)	176.26 (29.66)	.181	.218
Previous antiplatelet therapy	39 (28.5%)	92 (24.0%)	.304	98 (29.1%)	194 (22.2%)	.013	.647
Blend sign, present	33 (24.1%)	48 (12.5%)	.002	79 (23.4%)	89 (10.2%)	< .001	.362
Black hole sign, present	30 (21.9%)	49 (12.8%)	.013	77 (22.8%)	133 (15.2%)	.002	.265
Hypodensities, present	50 (36.5%)	84 (21.9%)	.001	134 (39.8%)	209 (23.9%)	< .001	.266
Island sign, present	16 (11.7%)	19 (4.9%)	.010	37 (11.0%)	51 (5.8%)	.003	.683

**Table 2:** Patient characteristics for the training and testing datasets with respect to functional outcome. Data are number (%), mean (SD), or median (IQR). ^‡^*p* value between testing and training datasets

	Testing dataset (*N* = 517)			Training dataset (*N* = 1202)			
	Poor functional outcome	Good functional outcome	*p* value	Poor functional outcome	Good functional outcome	*p* value	*p* value^‡^
Number	258 (49.9%)	259 (50.1%)		643 (53.5%)	559 (46.5%)		
Age, years	72.55 (12.30)	65.29 (12.75)	< .001	73.10 (12.80)	64.08 (13.52)	< .001	.989
Gender, male	128 (49.6%)	164 (63.3%)	.002	319 (49.6%)	353 (63.1%)	< .001	.826
Treatment allocation, placebo	130 (50.4%)	129 (49.8%)	.930	320 (49.8%)	283 (50.6%)	.773	.979
Baseline volume, mL	20.20 (9.79–40.49)	7.27 (3.51–14.51)	< .001	18.78 (7.71–42.14)	7.50 (3.36–14.83)	< .001	.869
Onset to CT scan, hours	1.89 (1.33– 2.93)	2.00 (1.38– 3.05)	.387	1.92 (1.33– 2.75)	1.95 (1.37– 2.83)	.178	.497
Ultra-early haematoma growth, mL/h	10.40 (4.78–20.98)	4.09 (1.52–7.39)	< .001	9.65 (4.04–22.61)	3.58 (1.62–8.06)	< .001	.865
Systolic blood pressure, mm Hg	172.26 (28.63)	174.84 (29.81)	.316	174.93 (29.14)	176.08 (29.24)	.497	.214
Previous antiplatelet therapy	79 (30.6%)	52 (20.1%)	.006	195 (30.3%)	97 (17.4%)	< .001	.644
Blend sign, present	49 (19.0%)	31 (12.0%)	.029	105 (16.3%)	62 (11.1%)	.009	.392
Black hole sign, present	51 (19.8%)	27 (10.4%)	.003	147 (22.9%)	61 (10.9%)	< .001	.258
Hypodensities, present	87 (33.7%)	46 (17.8%)	< .001	233 (36.2%)	107 (19.1%)	< .001	.276
Island sign, present	32 (12.4%)	3 (1.2%)	< .001	79 (12.3%)	9 (1.6%)	< .001	.684

Regarding differences within the training and testing sets, we observed that baseline haemorrhage volume was significantly higher (*p* < .001) for participants who developed HE and for those with poor functional outcome in both sets. This difference remains significant if we test on the whole population sample (*p* < .001). For treatment allocation, we observed a difference that is not statistically significant between participants with and without HE on both sets. However, this difference became significant when tested on the whole population sample (*p* = .018). We observed no statistical difference in poor functional outcome in the training and testing sets, nor when tested on the whole population sample (all *p* > .05).

### Effect of feature harmonisation

**Fig. 4:**
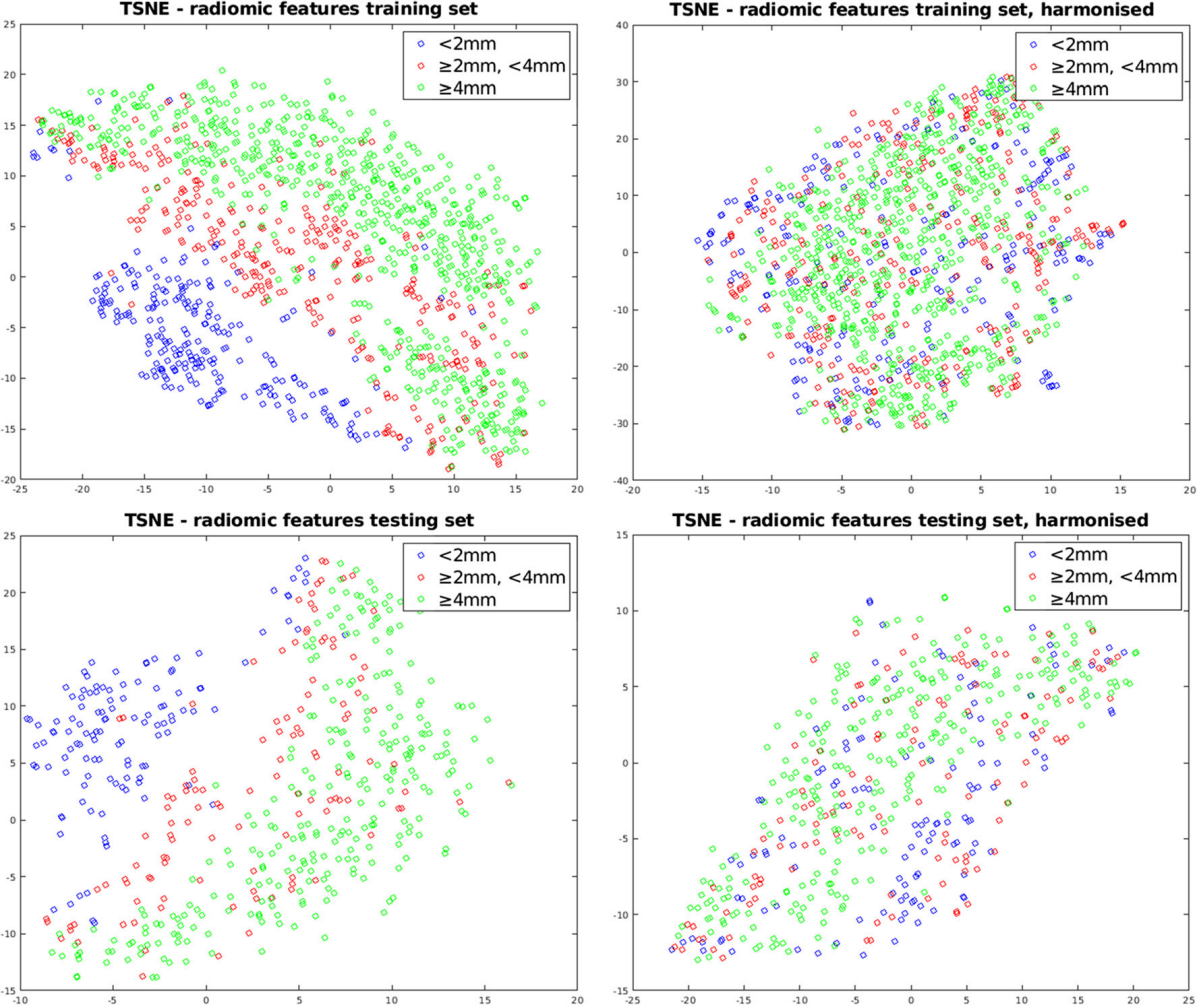
TSNE visualisations of standardised training and testing radiomics feature vectors for each of the 3 harmonisation batches. Each point represents a feature vector for one subject. The left column corresponds to subject radiomics feature vectors pre-harmonisation and the right column corresponds to subject radiomics feature vectors post-harmonisation

To show the effect of feature harmonisation, we computed a 2-dimensional t-SNE manifold [33] over the standardised feature vectors of each subject for both the training and testing sets (Fig. 4). We observed that prior to harmonisation, there were three clusters corresponding to each of the harmonisation batches on both sets. This demonstrates the strong impact of slice thickness on the values of computed radiomics-based features. After harmonisation, this influence was eliminated.

### Performance of generalised linear models

**Fig. 5:**
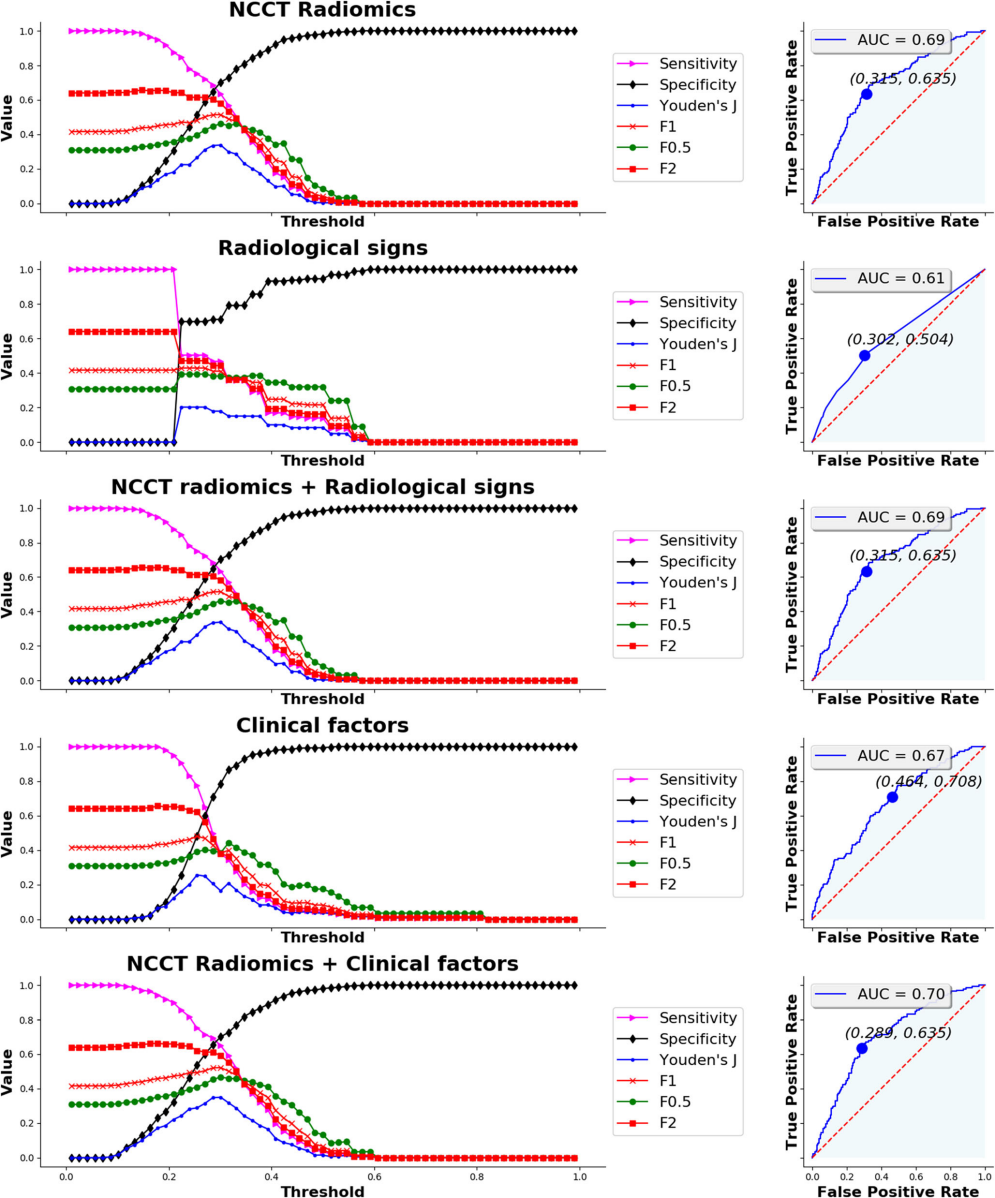
Threshold analysis for sensitivity, specificity, Youden’s index, F1 score, F0.5 score, and F2 score (left column) and ROC curve (right column) for the five prediction models of haematoma expansion (radiomics, radiological signs, radiomics and signs combined, clinical factors, and radiomics and clinical factors combined). Optimal threshold criterion was maximal Youden’s index

**Fig. 6:**
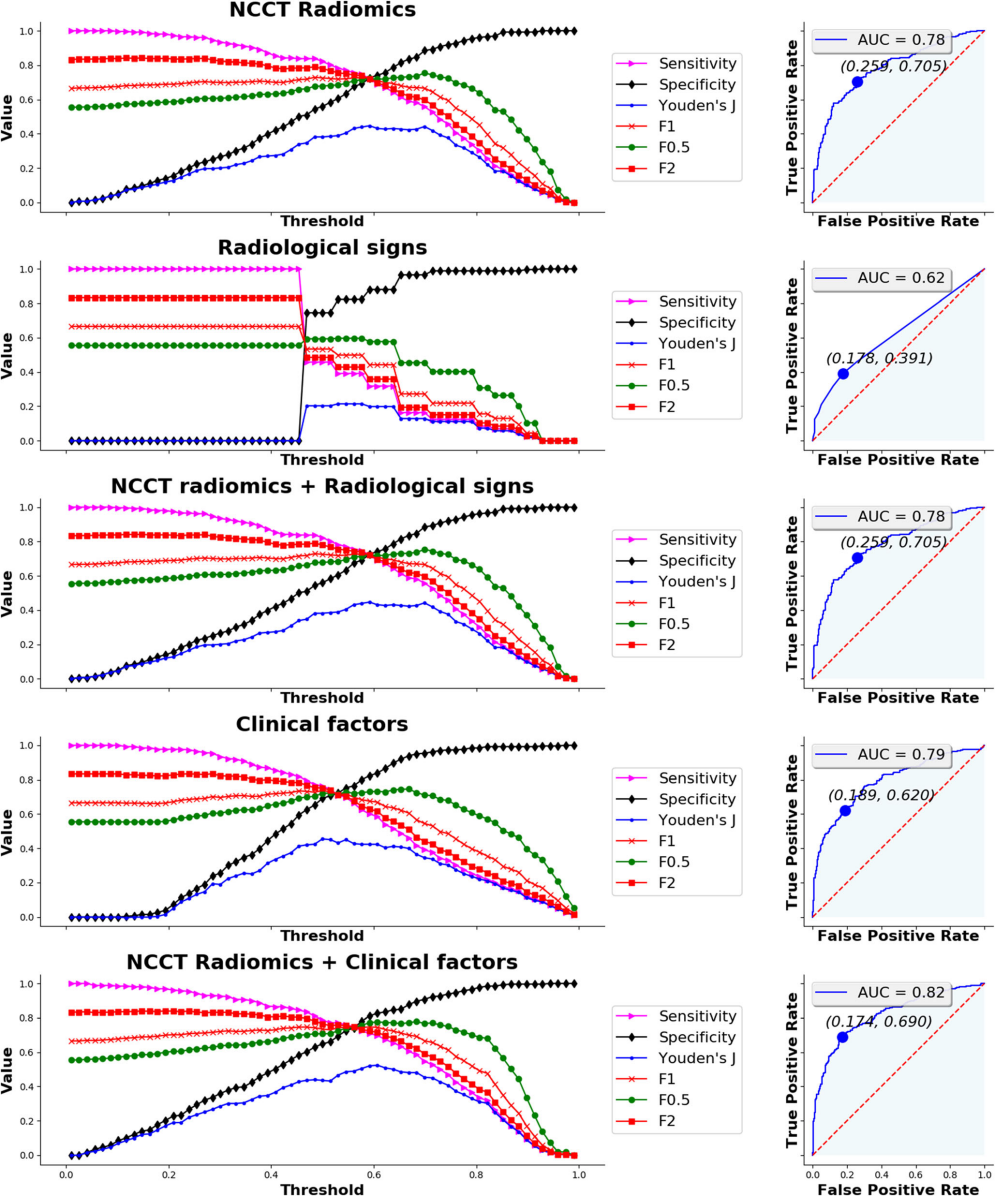
Threshold analysis for sensitivity, specificity, Youden’s index, F1 score, F0.5 score, and F2 score (left column) and ROC curve (right column) for the three prediction models of poor functional (radiomics, radiological signs, radiomics and signs combined, clinical factors, and radiomics and clinical factors combined). Optimal threshold criterion was maximal Youden’s index

Threshold analysis and ROC curves summarising the prediction performance of the optimal models on the training set using NCCT radiomics-based features, radiological signs, clinical factors, and combined models are depicted in Fig. 5 for HE and Fig. 6 for poor functional outcome. Individual AUC, sensitivity, specificity, positive predictive value (PPV), negative predictive value (NPV), and prevalence for both the training and testing sets are reported in Table 3. For each optimal model, we chose the threshold probability in the training set such that Youden’s index defined as *sensitivity* + *specificity* − 1 is maximised. The same threshold was then used on the testing set.

**Table 3:** Performance table of all models, both on the testing and training sets. *NCCT*, noncontrast computed tomography; *AUC*, area under the receiver operating characteristic curve; *PPV*, positive predictive value; *NPV*, negative predictive value

			Testing dataset						Training dataset					
		Threshold	AUC (95% CI)	Sensitivity (95% CI)	Specificity (95% CI)	PPV (95% CI)	NPV (95% CI)	Prevalence (95% CI)	AUC (95% CI)	Sensitivity (95% CI)	Specificity (95% CI)	PPV (95% CI)	NPV (95% CI)	Prevalence (95% CI)
Haematoma expansion	NCCT radiomics	0.2986	0.693 (0.638–0.747)	0.635 (0.554–0.716)	0.690 (0.644–0.736)	0.422 (0.355–0.49)	0.841 (0.801–0.882)	0.263 (0.225–0.301)	0.681 (0.646–0.716)	0.602 (0.550–0.655)	0.672 (0.640–0.703)	0.414 (0.371–0.458)	0.814 (0.786–0.843)	0.278 (0.253–0.304)
	Radiological signs	0.2793	0.609 (0.552–0.665)	0.467 (0.384–0.551)	0.711 (0.666–0.756)	0.366 (0.294–0.437)	0.789 (0.746–0.832)	0.263 (0.225–0.301)	0.620 (0.584–0.656)	0.507 (0.454–0.561)	0.700 (0.670–0.731)	0.395 (0.349–0.441)	0.787 (0.758–0.815)	0.278 (0.253–0.304)
	NCCT radiomics + radiological signs	0.2986	0.693 (0.638–0.747)	0.635 (0.554–0.716)	0.69 (0.644–0.736)	0.422 (0.355–0.49)	0.841 (0.801–0.882)	0.263 (0.225–0.301)	0.681 (0.646–0.716)	0.602 (0.550–0.655)	0.672 (0.640–0.703)	0.414 (0.371–0.458)	0.814 (0.786–0.843)	0.278 (0.253–0.304)
	Clinical factors	0.3101	0.668 (0.613–0.723)	0.350 (0.270–0.430)	0.839 (0.802–0.875)	0.436 (0.344–0.529)	0.783 (0.744–0.823)	0.263 (0.225–0.301)	0.637 (0.601–0.673)	0.368 (0.316–0.419)	0.835 (0.811–0.86)	0.463 (0.403–0.522)	0.774 (0.747–0.801)	0.278 (0.253–0.304)
	NCCT radiomics + clinical factors	0.3073	0.704 (0.651–0.758)	0.650 (0.570–0.730)	0.711 (0.666–0.756)	0.445 (0.376–0.514)	0.85 (0.811–0.889)	0.263 (0.225–0.301)	0.690 (0.655–0.725)	0.588 (0.535–0.640)	0.706 (0.676–0.736)	0.435 (0.390–0.481)	0.816 (0.789–0.844)	0.278 (0.253–0.304)
Poor functional outcome	NCCT radiomics	0.6035	0.783 (0.744–0.823)	0.698 (0.642–0.754)	0.741 (0.688–0.795)	0.729 (0.673–0.784)	0.711 (0.657–0.765)	0.499 (0.456–0.542)	0.814 (0.790–0.834)	0.644 (0.607–0.681)	0.844 (0.814–0.874)	0.826 (0.793–0.860)	0.673 (0.639–0.708)	0.535 (0.507–0.563)
	Radiological signs	0.5911	0.621 (0.573–0.669)	0.318 (0.261–0.375)	0.880 (0.841–0.920)	0.726 (0.643–0.808)	0.564 (0.516–0.613)	0.499 (0.456–0.542)	0.612 (0.580–0.643)	0.327 (0.290–0.363)	0.864 (0.836–0.892)	0.734 (0.683–0.785)	0.527 (0.495–0.560)	0.535 0.507–0.563)
	NCCT radiomics + radiological signs	0.6035	0.783 (0.744–0.823)	0.698 (0.642–0.754)	0.741 (0.688–0.795)	0.729 (0.673–0.784)	0.711 (0.657–0.765)	0.499 (0.456–0.542)	0.814 (0.790–0.834)	0.644 (0.607–0.681)	0.844 (0.814–0.874)	0.826 (0.793–0.860)	0.673 (0.639–0.708)	0.535 (0.507–0.563)
	Clinical factors	0.5845	0.789 (0.750–0.828)	0.620 (0.561–0.679)	0.815 (0.767–0.862)	0.769 (0.712–0.826)	0.683 (0.631–0.735)	0.499 (0.456–0.542)	0.777 (0.751–0.803)	0.596 (0.558–0.634)	0.818 (0.786–0.850)	0.790 (0.753–0.826)	0.637 (0.602–0.673)	0.535 (0.507–0.563)
	NCCT radiomics + clinical factors	0.6094	0.818 (0.781–0.854)	0.694 (0.638–0.750)	0.826 (0.780–0.872)	0.799 (0.747–0.852)	0.730 (0.68–0.781)	0.499 (0.456–0.542)	0.835 (0.813–0.858)	0.663 (0.626–0.699)	0.852 (0.822–0.881)	0.837 (0.805–0.869)	0.687 (0.652–0.721)	0.535 (0.507–0.563)

For prediction of haematoma expansion, a moderate performance was observed when using NCCT radiomics-based features, with testing set AUC of 0.693 (95% CI, 0.638–0.747), sensitivity of 0.635 (95% CI, 0.554–0.716), specificity of 0.690 (95% CI, 0.644–0.736), PPV of 0.422 (95% CI, 0.355–0.49), and NPV of 0.841 (95% CI, 0.801–0.882). The rather low PPV can be partially explained by an imbalance of the positive and negative classes, as prevalence was 26.3% (95% CI, 22.5–30.1%). The most important feature in the GLM was “*LoG-35 interquartile range*” (Table 4), which highlights that haematoma heterogeneity (as measured by Laplacian of Gaussian-based features) is relevant for prediction. The performance of radiological signs was considerably lower (Table 3) due to sharp reductions in sensitivity and AUC on both sets; however, specificities were mildly higher. Moreover, the optimal model for prediction using radiomics-based features and radiological signs combined was identical to the optimal model using radiomics-based features alone, as all the coefficients for radiological signs were shrunk to zero by elastic-net training. Also, the optimal model using demographic and clinical factors yielded slightly lower performance than the one using radiomics-based features, especially due to a sharp reduction in sensitivity to 0.35 (95% CI, 0.27–0.43). However, combining these factors with radiomics-based features boosted their performance, achieving an AUC of 0.704 (95% CI, 0.651–0.758) and an increased sensitivity of 0.65 (95% CI, 0.57–0.73). We found that time from onset to CT scan was the factor responsible for this improvement. Finally, treatment allocation was discarded by elastic net in all radiomics-based models, showing it had no effect on predictions.

**Table 4:** Ranking of radiomics-based features selected in elastic-net training in terms of their importance. Only features with an importance greater than 1% are shown. Standardised model coefficients are also provided in boldface (positive) and italics (negative)

	Haematoma expansion						Poor functional outcome					
	NCCT features			NCCT features + clinical factors			NCCT features			NCCT features + clinical factors		
	Feature name	Model coefficient	Importance percentage	Feature name	Model coefficient	Importance percentage	Feature name	Model coefficient	Importance percentage	Feature name	Model coefficient	Importance percentage
	(Intercept)	*− 1.00111*	-	(Intercept)	*−1.00828*	-	(Intercept)	**0.17692**	-	(Intercept)	**0.19116**	-
1	LoG-35 interquartile range	*−0.28305*	45.9%	LoG-35 interquartile range	*−0.28752*	38.1%	Perihaematomal oedema volume	**0.28676**	8.0%	Age	**0.65611**	20.5%
2	LoG-15 GLSZM grey level non-uniformity	**0.10061**	16.3%	Intensities GLSZM small zone emphasis	*−0.10982*	14.6%	LoG-35 variance	*−0.27735*	7.7%	LoG-35 variance	*−0.33578*	10.5%
3	Intensities GLSZM small zone emphasis	*−0.09417*	15.3%	Onset to CT scan	*−0.09544*	12.7%	Wavelet-HHH mean	**0.24911**	6.9%	Ultra-early haematoma growth	**0.26497**	8.3%
4	Intracerebral haemorrhage major axis length	**0.06405**	10.4%	LoG-15 GLSZM grey level non-uniformity	**0.09340**	12.4%	Wavelet-LLL mean	*−0.24302*	6.8%	Perihaematomal oedema volume	**0.17074**	5.3%
5	LoG-25 mean absolute deviation	*−0.02980*	4.8%	Intracerebral haemorrhage major axis length	**0.06263**	8.3%	Wavelet-HHH kurtosis	*−0.21109*	5.9%	Wavelet-HHH kurtosis	−*0.15967*	5.0%
6	LoG-25 kurtosis	**0.02967**	4.8%	LoG-25 kurtosis	**0.04722**	6.3%	Intracerebral haemorrhage sphericity	*−0.20476*	5.7%	LoG-25 GLSZM grey level non-uniformity	**0.12835**	4.0%
7	Intensities 10th percentile	**0.01588**	2.6%	Intensities 10th percentile	**0.03175**	4.2%	Intraventricular haemorrhage volume	**0.17809**	5.0%	Intensities 10th percentile	−*0.12659*	3.9%
8				LoG-25 mean absolute deviation	*−0.02238*	3.0%	LoG-25 GLSZM grey level non-uniformity	**0.16785**	4.7%	Intraventricular haemorrhage volume	**0.12149**	3.8%
9							Intracerebral haemorrhage flatness	**0.14927**	4.2%	Wavelet-HHH mean	**0.10174**	3.2%
10							Wavelet-LLH energy	*−0.14774*	4.1%	LoG-15 GLRLM long run low grey level emphasis	*−0.10067*	3.1%
11							LoG-05 GLSZM grey level variance	*−0.13732*	3.8%	Intracerebral haemorrhage flatness	**0.09718**	3.0%
12							Wavelet-HHL energy	**0.13382**	3.7%	Intracerebral haemorrhage sphericity	*−0.09118*	2.8%
13							Wavelet-LHL GLCM dissimilarity	**0.13070**	3.6%	LoG-05 GLSZM grey level variance	*−0.08755*	2.7%
14							Wavelet-LLL GLRLM long run low grey level emphasis	*−0.12947*	3.6%	Wavelet-LLL GLRLM long run low grey level emphasis	*−0.08652*	2.7%
15							Wavelet-LHL median	*−0.11564*	3.2%	Wavelet-LLH GLRLM long run low grey level emphasis	*−0.08570*	2.7%
16							LoG-15 GLRLM long run low grey level emphasis	*−0.07124*	2.0%	Wavelet-HHH NGTDM complexity	**0.07429**	2.3%
17							Wavelet-HHH NGTDM complexity	**0.05407**	1.5%	LoG-35 GLSZM small zone low grey level emphasis	*−0.06147*	1.9%
18							Wavelet-LHH median	**0.04914**	1.4%	Wavelet-LHL GLCM dissimilarity	**0.05893**	1.8%
19							Wavelet-HHL maximum	*−0.04642*	1.3%	LoG-35 NGTDM complexity	*−0.05137*	1.6%
20							Wavelet-LLH GLCM entropy	**0.04512**	1.3%	Wavelet-LHL GLSZM small zone high grey level emphasis	*−0.05110*	1.6%
21							Wavelet-HHH minimum	**0.04480**	1.2%	Wavelet-HHL GLRLM long run low grey level emphasis	*−0.05094*	1.6%
22							Wavelet-HHL GLRLM long run low grey level emphasis	*−0.04402*	1.2%	Wavelet-LHL median	*−0.04691*	1.5%
23							Wavelet-LLL minimum	*−0.03991*	1.1%	Wavelet-LLL GLRLM run length variance	*−0.04173*	1.3%
24							LoG-35 NGTDM complexity	*−0.03688*	1.0%	Wavelet-LLL minimum	*−0.03896*	1.2%
25							Wavelet-LHL GLSZM small zone high grey level emphasis	*−0.03675*	1.0%			

In the case of prediction of poor functional outcome using NCCT radiomics-based features, we observed a good performance, with testing set AUC of 0.783 (95% CI, 0.744–0.823), sensitivity of 0.698 (95% CI, 0.642–0.754), specificity of 0.741 (95% CI, 0.688–0.795), PPV of 0.729 (95% CI, 0.673–0.784), and NPV of 0.711 (95% CI, 0.657–0.765). Here, the balanced prevalence of 49.9% (95% CI, 45.6–54.2%) is reflected on balanced predictive values. Perihaematomal oedema volume was found as the most important factor for prediction, suggesting that inflammation may have an important effect on clinical outcome. Like the case of haematoma expansion, both sensitivity and AUC suffered a strong reduction using radiological signs compared to the model trained with NCCT radiomics-based features. Again, for the case of combined NCCT radiomics-based features and radiological signs, we obtained a model which was identical to the one trained using NCCT radiomics-based features alone. Moreover, demographic and clinical factors showed slightly better AUC than NCCT radiomics-based features, explained by an improved specificity. Again, by combining these factors and NCCT radiomics-based features, the best performance is achieved, with an AUC of 0.818 (95% CI, 0.781–0.854), mainly due to increased PPV and NPV of 0.799 (95% CI, 0.747–0.852) and 0.73 (95% CI, 0.68–0.781), respectively. The features accountable for this improvement were age and ultra-early haematoma growth. Lastly, treatment allocation had again no influence on the radiomics-based models and was discarded by elastic net.

## Discussion

We evaluated the predictive performance of NCCT radiomics-based features for haematoma expansion and poor functional outcome using generalised linear models on a large and heterogeneous sample. We also investigated the predictive performance of radiological signs and clinical factors, and their combination with radiomics-based features with the same type of models. This analysis evidenced that radiomics-based features have higher predictive performance compared to radiological signs and perform similarly to clinical factors. Moreover, this work showed that prediction performance is not improved by incorporating radiological signs, but it is boosted by the inclusion of demographic and clinical factors.

Since our analyses are based on binary outcomes, haematoma expansion and the modified Rankin scale were dichotomised. Our choice of dichotomisation for haematoma expansion was identical to that of the TICH-2 trial [20] and has also been used in several retrospective analyses [10, 34–36]. Our choice for the modified Rankin scale is based on the observation that intracerebral haemorrhage is in general a severe type of stroke and a score of 3 or less would still be considered a “good” outcome. This choice has also been previously made for the primary analysis of large clinical trials of ICH, such as STICH [37] and CLEAR III [38].

Our reported diagnostic performance of radiological signs was not identical to a previous work using data from the TICH-2 clinical trial [13]. The main sources of difference were that our study included fewer participants and that we performed the analysis using the combined set of signs as predictor features simultaneously, rather than testing each one individually. Nevertheless, our results are consistent with this previous report in terms of the relatively low sensitivity showed by these signs.

In addition to their poor sensitivity, radiological signs are qualitative measures with subjective definitions and are therefore susceptible to inter- and intra-rater variability. On the other hand, the proposed radiomics-based generalised linear model provides a quantitative and consistent way of predicting HE and poor functional outcome, yielding better sensitivity and AUC. However, despite the performance of our radiomics approach, it is not currently available for real-time application and would require significant further development and validation for clinical use.

Many important variables selected by our radiomics-based model are consistent with previous findings. Amongst them are features based on Laplacian of Gaussian filters, which are indicative of haematoma heterogeneity and have good predictive value for its expansion [39], and extent of perihaematomal oedema, which has a predictive association with poor functional outcome [40, 41]. Also, our observation that the addition of the time from symptom onset to baseline scan to our radiomics-based model improves HE prediction is consistent with the meta-analysis by Al-Shahi Salman et al [14]. Finally, the conclusion that incorporating age and ultra-early haematoma growth as factors substantially improves functional outcome prediction has strong support in prior studies [30, 42, 43].

An important aspect of constructing a NCCT radiomics-based model for prediction is the harmonisation of the extracted raw features. This is especially crucial in the context of data coming from multicentre studies such as TICH-2, where the stability of the radiomics features can be severely affected by differences in imaging protocols [44]. Our investigation suggests that slice thickness is a very important factor to consider for harmonisation, which is in agreement with previous findings [45]. However, there are additional relevant sources of difference (for which we had no exhaustive information) that could have been considered such as scan type (helical/axial), slice gap, reconstruction kernel, field of view, tube voltage, and milliamperage [46]. Future radiomics studies should take all these sources of variability into account to achieve optimal harmonisation.

A relevant observation is that our model does not reach the same predictive performance for HE as recent studies using radiomics-based linear models [18, 19] that show substantially better performance. This may be due to those studies being performed on significantly smaller samples from 4 or fewer centres which may have much lower variability in terms of CT parameters. This highlights the challenges that radiomics-based models face when dealing with data that better reflects the “real-world” variability present in multicentre studies. In order to provide a reliable tool for research and clinical application, it is also essential to tackle the current challenge of reproducibility in advanced clinical imaging. In radiomics, potential sources of variability arise from image pre- and post-processing, discrepancies in the way the segmentation of regions of interest is performed, and differences in the number and type of radiomics computed. Standardisation efforts such as IBSI [22] are a step in the right direction and should be adopted in future radiomics studies. Furthermore, we have found beneficial to perform post-harmonisation of discrepant features when protocol homologation amongst many centres is not feasible or practical, like in the case of our study.

Finally, this is a first approach using elastic net, where the linear contributions of each feature can be assessed in a straightforward manner. Other machine learning approaches, such as random forest or deep learning, can be investigated to improve the detection of non-linear interactions between variables and potentially improve prediction performance. For example, a comparison of different classifiers similar to the one performed by Li et al [17] may be pursued, but in a much larger sample.

In conclusion, we showed that models using NCCT radiomics-based features outperform models using radiological signs or clinical factors. However, we found that incorporating demographic and clinical factors into a radiomics-based model substantially improves the prediction. These results suggest that radiomics-based models, with added demographic and clinical factors if available, may be of prognostic value in people with ICH. Hence, they could be incorporated into therapeutic trials to aid selection of those at risk of haematoma expansion or poor functional outcome once further work has been performed to address the challenges around predictive accuracy and variability of radiomics features in multicentre studies.

## Supplementary Information

ESM 1 (DOCX 79 kb)

## Abbreviations

AUC: Area under the receiver operating characteristic curve
CTA: Computed tomography angiography
GLM: Generalised linear model
HE: Haematoma expansion
ICH: Intracerebral haemorrhage
LASSO: Least absolute shrinkage and selection operator
NCCT: Noncontrast computed tomography
NPV: Negative predictive value
PPV: Positive predictive value
TICH-2: Tranexamic acid for intracerebral haemorrhage 2
TSNE: t-Distributed stochastic neighbour embedding

## Declarations

### Guarantor

The scientific guarantor of this publication is Dr. Rob Dineen.

### Statistics and biometry

No complex statistical methods were necessary for this paper.

### Study subjects or cohorts overlap

Some study subjects or cohorts have been previously reported in *Law ZK, Ali A, Krishnan K, et al (2019) Noncontrast computed tomography signs as predictors of hematoma expansion, clinical outcome, and response to tranexamic acid in acute intracerebral hemorrhage. Stroke 121–128.* https://doi.org/10/ggc8d5*.*

### Methodology

• retrospective

• diagnostic or prognostic study

• multicenter study
