# Supplementary material for: Quantitative CT radiomics-based models for prediction of haematoma expansion and poor functional outcome in primary intracerebral haemorrhage
Source: Eur Radiol. 2021 Apr 16;31(10):7945–59. doi: 10.1007/s00330-021-07826-9 (PMC8452575; doi:10.1007/s00330-021-07826-9)
Supplement: Supplementary file 1 — (DOCX 79 kb) [file 330_2021_7826_MOESM1_ESM.docx]

Supplementary Materials

Radiomics-based features

We compute a total of 752 NCCT radiomics-based features from manual annotations of intracerebral haemorrhage. They can be categorised as 11 shape-based, 17 first-order features (see Algorithm S1 and Algorithm S2), and 40 texture factors corresponding to 9 grey level Co-occurrence Matrix (GLCM) features, 13 grey level Size zone Matrix (GLSZM) features, 13 grey level run Length Matrix (GLRLM) features, and 5 Neighbouring grey Tone Difference Matrix (NGTDM) features. All textural matrices were computed using uniform quantisation. Images were resampled to 1mm isotropic voxel size using nearest neighbour interpolation for volumetric annotations and cubic interpolation for NCCT scans. Filtered versions of the resampled scans were also computed using a Laplacian of Gaussian (LoG) filter at 4 different levels (3x3x3 voxels kernel with σ=0.5; 7x7x7 voxels kernel with σ=1.5; 11x11x11 voxels kernel with σ=2.5; and 13x13x13 voxels kernel with σ=3.5) and a *sym8* Wavelet filter with 8 channels (LLL, LLH, LHL, LHH, HLL, HLH, HHL, and HHH). Shape features are computed directly from the resampled manual annotations, whereas first-order and texture factors are computed from the region of interest defined by these annotations on the NCCT scans (using 50 grey levels for histogram computation and uniform quantisation) and on their LoG-and Wavelet filtered versions (using 100 grey levels for histogram computation and uniform quantisation) . Therefore, given that we have 1 NCCT image, 4 LoG-filtered images and 8 Wavelet filtered images, the aggregated number of features is 11 + 17*(1+4+8) + 40*(1+4+8) = 752. By adding perihaematomal oedema volume and intraventricular haemorrhage volume as additional factors, we obtain the final set of 754 features. A summary of all types of radiomics-based features is shown in Table S1. The set of unrelated features used in the generalised linear model construction is summarised in Table S2.

Grid optimisation over blending hyperparameter α

We performed an exhaustive search-grid optimisation procedure to find the elastic-net blending hyperparameter $\alpha$ that yields the optimal model with the highest cross-validation AUC (see Table S3). We observed that there was no meaningful difference in performance between different $\alpha$ values.

Radiomics Quality Score evaluation

A summary of the Radiomics Quality Score evaluation of the proposed study is provided on Table S4. Note that, despite TICH-2 being a registered prospective clinical trial, it was not designed as a radiomics trial which substantially affected the score.

function Fvec = compute_shape_features(img, isotropic_voxel_size_mm)

mask = ~isnan(img);

stats = regionprops3(mask, {'Volume', 'SurfaceArea', 'EigenValues'});

V = stats.Volume * (isotropic_voxel_size_mm^3) / 1000; %volume in cm3

A = stats.SurfaceArea * (isotropic_voxel_size_mm^2) / 100; %area in cm2

L = sort(stats.EigenValues{1}, 'ascend');

L_least = L(1);

L_minor = L(2);

L_major = L(3);

Fvec = zeros(1, 11);

Fvec(1) = V / sqrt(pi * (A^3)); %compactness1

Fvec(2) = 36 * pi * ((V^2) / (A^3)); %compactness2

Fvec(3) = V; %volume

Fvec(4) = A; %surface area

Fvec(5) = A / V; %surface to volume ratio

Fvec(6) = nthroot(36 * pi * (V^2), 3) / A; %sphericity

Fvec(7) = 4 * L_major; %major axis length

Fvec(8) = 4 * L_minor; %minor axis length

Fvec(9) = 4 * L_least; %least axis length

Fvec(10) = sqrt(L_minor / L_major); %elongation

Fvec(11) = sqrt(L_least / L_major); %flatness

end

Algorithm S2: MATLAB code snippet for the computation of shape features.

Algorithm S1: MATLAB code snippet for the computation of first-order features.

function Fvec = compute_first_order_features(img, levels)

mask = ~isnan(img);

N = sum(mask(:));

I = img(mask(:));

p = histcounts(I, levels);

p = p ./ N;

percentile_vals = prctile(I, [10 25 75 90]);

mask_robust = mask & (img >= percentile_vals(1)) & (img <= percentile_vals(4));

N_robust = sum(mask_robust(:));

I_robust = img(mask_robust(:));

Fvec = zeros(1, 17);

Fvec(1) = percentile_vals(1); %10th precentile

Fvec(2) = percentile_vals(4); %90th precentile

Fvec(3) = sumsqr(I); %energy

Fvec(4) = -sum(p .* log2(p + eps)); %entropy

Fvec(5) = percentile_vals(3) - percentile_vals(2); %interquartile range (75th minus 25th percentile)

Fvec(6) = kurtosis(I); %kurtosis

Fvec(7) = max(I); %maximum

Fvec(8) = (1/N) * sum(abs(bsxfun(@minus, I, mean(I)))); %mean absolute deviation

Fvec(9) = mean(I); %mean

Fvec(10) = median(I); %median

Fvec(11) = min(I); %minimum

Fvec(12) = max(I) - min(I); %range

Fvec(13) = (1/N_robust) * sum(abs(bsxfun(@minus, I_robust, mean(I_robust)))); %robust mean absolute deviation

Fvec(14) = sqrt((1/N) * sumsqr(I)); %root mean squared

Fvec(15) = skewness(I); %skewness

Fvec(16) = sumsqr(p); %uniformity

Fvec(17) = var(I); %variance

end

| **First order features** | |
| --- | --- |
|  | 10^th^ percentile  90^th^ percentile  Energy  Entropy  Interquartile range  Kurtosis  Maximum  Mean absolute deviation  Mean  Median  Minimum  Range  Robust mean absolute deviation  Root mean squared  Skewness  Uniformity  Variance |
| **Shape features** | |
|  | Compactness 1  Compactness 2  Volume  Surface area  Surface to volume ratio  Sphericity  Major axis length  Minor axis length  Least axis length  Elongation  Flatness |
| **Textural features** | |
| Grey Level Co-occurrence Matrix (GLCM) | Dissimilarity  Auto correlation  Energy  Contrast  Correlation  Homogeneity  Variance  Sum average  Entropy |
| Grey Level Size Zone Matrix (GLSZM) | Small zone emphasis  Large zone emphasis  Grey level non-uniformity  Zone size non-uniformity  Zone percentage  Low grey level zone emphasis  High grey level zone emphasis  Small zone low grey level emphasis  Small zone high grey level emphasis  Large zone low grey level emphasis  Large zone high grey level emphasis  Grey level variance  Zone size variance |
| Grey Level Run Length Matrix (GLRLM) | Short run emphasis  Long run emphasis  Grey level non-uniformity  Run length non-uniformity  Run percentage  Low grey level run emphasis  High grey level run emphasis  Short run low grey level emphasis  Short run high grey level emphasis  Long run low grey level emphasis  Long run high grey level emphasis  Grey level variance  Run length variance |
| Neighbouring Grey Tone Difference Matrix (NGTDM) | Coarseness  Contrast  Busyness  Complexity  Strength |
| **Other shape features** | |
|  | Perihaematomal oedema volume  Intraventricular haemorrhage volume |

Table S1: Summary of extracted features.

| **Unrelated features** |
| --- |
| Perihaematomal oedema volume  Intraventricular haemorrhage volume  Intracerebral haemorrhage surface to volume ratio  Intracerebral haemorrhage sphericity  Intracerebral haemorrhage major axis length  Intracerebral haemorrhage elongation  Intracerebral haemorrhage flatness  Intensities 10th percentile  Intensities kurtosis  Intensities range  Intensities GLCM correlation  Intensities GLCM variance  Intensities GLCM entropy  Intensities GLRLM long run low grey level emphasis  Intensities GLRLM long run high grey level emphasis  Intensities GLRLM grey level variance  Intensities GLRLM run length variance  Intensities GLSZM small zone emphasis  Intensities GLSZM grey level non-uniformity  Intensities GLSZM low grey level zone emphasis  Intensities GLSZM large zone low grey level emphasis  Intensities GLSZM grey level variance  Intensities GLSZM zone size variance  Intensities NGTDM contrast  Intensities NGTDM complexity  LoG-05 90th percentile  LoG-05 kurtosis  LoG-05 maximum  LoG-05 median  LoG-05 minimum  LoG-05 range  LoG-05 skewness  LoG-05 variance  LoG-05 GLCM dissimilarity  LoG-05 GLCM correlation  LoG-05 GLRLM short run high grey level emphasis  LoG-05 GLRLM long run low grey level emphasis  LoG-05 GLRLM grey level variance  LoG-05 GLRLM run length variance  LoG-05 GLSZM large zone emphasis  LoG-05 GLSZM grey level variance  LoG-05 NGTDM complexity  LoG-15 90th percentile  LoG-15 kurtosis  LoG-15 maximum  LoG-15 minimum  LoG-15 range  LoG-15 root mean squared  LoG-15 skewness  LoG-15 variance  LoG-15 GLCM dissimilarity  LoG-15 GLCM correlation  LoG-15 GLRLM short run high grey level emphasis  LoG-15 GLRLM long run low grey level emphasis  LoG-15 GLRLM grey level variance  LoG-15 GLRLM run length variance  LoG-15 GLSZM grey level non-uniformity  LoG-15 GLSZM large zone low grey level emphasis  LoG-15 GLSZM large zone high grey level emphasis  LoG-15 GLSZM grey level variance  LoG-15 NGTDM complexity  LoG-25 kurtosis  LoG-25 mean absolute deviation  LoG-25 minimum  LoG-25 range  LoG-25 skewness  LoG-25 GLCM correlation  LoG-25 GLCM entropy  LoG-25 GLRLM long run low grey level emphasis  LoG-25 GLRLM grey level variance  LoG-25 GLRLM run length variance  LoG-25 GLSZM large zone emphasis  LoG-25 GLSZM grey level non-uniformity  LoG-35 interquartile range  LoG-35 kurtosis  LoG-35 maximum  LoG-35 median  LoG-35 minimum  LoG-35 range  LoG-35 root mean squared  LoG-35 skewness  LoG-35 variance  LoG-35 GLRLM long run low grey level emphasis  LoG-35 GLRLM grey level variance  LoG-35 GLRLM run length variance  LoG-35 GLSZM grey level non-uniformity  LoG-35 GLSZM high grey level zone emphasis  LoG-35 GLSZM small zone low grey level emphasis  LoG-35 GLSZM large zone high grey level emphasis  LoG-35 GLSZM grey level variance  LoG-35 NGTDM complexity  Wavelet-LLL maximum  Wavelet-LLL mean  Wavelet-LLL minimum  Wavelet-LLL skewness  Wavelet-LLL variance  Wavelet-LLL GLRLM grey level non-uniformity  Wavelet-LLL GLRLM long run low grey level emphasis  Wavelet-LLL GLRLM grey level variance  Wavelet-LLL GLRLM run length variance  Wavelet-LLL GLSZM large zone low grey level emphasis  Wavelet-LLL GLSZM large zone high grey level emphasis  Wavelet-LLL NGTDM complexity  Wavelet-LLH energy  Wavelet-LLH kurtosis  Wavelet-LLH maximum  Wavelet-LLH mean  Wavelet-LLH median  Wavelet-LLH root mean squared  Wavelet-LLH skewness  Wavelet-LLH variance  Wavelet-LLH GLCM dissimilarity  Wavelet-LLH GLCM auto correlation  Wavelet-LLH GLCM correlation  Wavelet-LLH GLCM entropy  Wavelet-LLH GLRLM long run low grey level emphasis  Wavelet-LLH GLRLM grey level variance  Wavelet-LLH GLRLM run length variance  Wavelet-LLH GLSZM small zone high grey level emphasis  Wavelet-LLH GLSZM large zone high grey level emphasis  Wavelet-LLH GLSZM grey level variance  Wavelet-LLH NGTDM complexity  Wavelet-LHL kurtosis  Wavelet-LHL maximum  Wavelet-LHL mean  Wavelet-LHL median  Wavelet-LHL skewness  Wavelet-LHL variance  Wavelet-LHL GLCM dissimilarity  Wavelet-LHL GLCM correlation  Wavelet-LHL GLRLM long run low grey level emphasis  Wavelet-LHL GLRLM long run high grey level emphasis  Wavelet-LHL GLRLM grey level variance  Wavelet-LHL GLRLM run length variance  Wavelet-LHL GLSZM large zone emphasis  Wavelet-LHL GLSZM small zone high grey level emphasis  Wavelet-LHL GLSZM grey level variance  Wavelet-LHL NGTDM complexity  Wavelet-LHH kurtosis  Wavelet-LHH maximum  Wavelet-LHH mean  Wavelet-LHH median  Wavelet-LHH robust mean absolute deviation  Wavelet-LHH skewness  Wavelet-LHH GLCM correlation  Wavelet-LHH GLRLM long run low grey level emphasis  Wavelet-LHH GLRLM long run high grey level emphasis  Wavelet-LHH GLRLM grey level variance  Wavelet-LHH GLRLM run length variance  Wavelet-LHH GLSZM small zone high grey level emphasis  Wavelet-LHH GLSZM large zone high grey level emphasis  Wavelet-LHH GLSZM grey level variance  Wavelet-LHH NGTDM complexity  Wavelet-HLL kurtosis  Wavelet-HLL maximum  Wavelet-HLL mean  Wavelet-HLL median  Wavelet-HLL skewness  Wavelet-HLL variance  Wavelet-HLL GLCM dissimilarity  Wavelet-HLL GLCM correlation  Wavelet-HLL GLRLM long run low grey level emphasis  Wavelet-HLL GLRLM long run high grey level emphasis  Wavelet-HLL GLRLM grey level variance  Wavelet-HLL GLRLM run length variance  Wavelet-HLL GLSZM small zone high grey level emphasis  Wavelet-HLL GLSZM large zone low grey level emphasis  Wavelet-HLL GLSZM large zone high grey level emphasis  Wavelet-HLL GLSZM grey level variance  Wavelet-HLL NGTDM complexity  Wavelet-HLH kurtosis  Wavelet-HLH mean  Wavelet-HLH median  Wavelet-HLH range  Wavelet-HLH skewness  Wavelet-HLH variance  Wavelet-HLH GLCM correlation  Wavelet-HLH GLRLM long run low grey level emphasis  Wavelet-HLH GLRLM long run high grey level emphasis  Wavelet-HLH GLRLM grey level variance  Wavelet-HLH GLRLM run length variance  Wavelet-HLH GLSZM large zone emphasis  Wavelet-HLH GLSZM zone size non-uniformity  Wavelet-HLH GLSZM small zone high grey level emphasis  Wavelet-HLH GLSZM grey level variance  Wavelet-HLH NGTDM complexity  Wavelet-HHL energy  Wavelet-HHL kurtosis  Wavelet-HHL maximum  Wavelet-HHL mean  Wavelet-HHL median  Wavelet-HHL root mean squared  Wavelet-HHL skewness  Wavelet-HHL GLCM dissimilarity  Wavelet-HHL GLCM correlation  Wavelet-HHL GLRLM long run low grey level emphasis  Wavelet-HHL GLRLM long run high grey level emphasis  Wavelet-HHL GLRLM grey level variance  Wavelet-HHL GLRLM run length variance  Wavelet-HHL GLSZM large zone emphasis  Wavelet-HHL GLSZM small zone high grey level emphasis  Wavelet-HHL GLSZM grey level variance  Wavelet-HHL NGTDM complexity  Wavelet-HHH kurtosis  Wavelet-HHH mean  Wavelet-HHH median  Wavelet-HHH minimum  Wavelet-HHH skewness  Wavelet-HHH GLCM correlation  Wavelet-HHH GLRLM long run low grey level emphasis  Wavelet-HHH GLRLM long run high grey level emphasis  Wavelet-HHH GLRLM grey level variance  Wavelet-HHH GLRLM run length variance  Wavelet-HHH GLSZM large zone emphasis  Wavelet-HHH GLSZM zone size non-uniformity  Wavelet-HHH GLSZM small zone high grey level emphasis  Wavelet-HHH GLSZM grey level variance  Wavelet-HHH NGTDM complexity |

Table S2: List of unrelated features used in model construction.

|  |  | Haematoma expansion | | Poor functional outcome | |
| --- | --- | --- | --- | --- | --- |
|  |  | **α value** | **Cross-validation AUC** | **α value** | **Cross-validation AUC** |
| NCCT radiomics | 1 | 1.0 | 0.6643 | 1.0 | 0.7769 |
|  | 2 | 0.9 | 0.6640 | 0.6 | 0.7769 |
|  | 3 | 0.8 | 0.6634 | 0.8 | 0.7769 |
|  | 4 | 0.7 | 0.6629 | 0.9 | 0.7769 |
|  | 5 | 0.6 | 0.6627 | 0.3 | 0.7768 |
|  | 6 | 0.5 | 0.6623 | 0.7 | 0.7768 |
|  | 7 | 0.4 | 0.6621 | 0.5 | 0.7768 |
|  | 8 | 0.3 | 0.6608 | 0.4 | 0.7767 |
|  | 9 | 0.2 | 0.6606 | 0.2 | 0.7766 |
|  | 10 | 0.1 | 0.6593 | 0.1 | 0.7757 |
|  | 11 | 0.0 | 0.6508 | 0.0 | 0.7724 |
| Radiological signs | 1 | 0.0 | 0.5988 | 0.0 | 0.5797 |
|  | 2 | 0.1 | 0.5985 | 0.1 | 0.5796 |
|  | 3 | 0.2 | 0.5982 | 0.2 | 0.5795 |
|  | 4 | 0.3 | 0.5980 | 0.4 | 0.5794 |
|  | 5 | 0.7 | 0.5957 | 0.6 | 0.5794 |
|  | 6 | 0.6 | 0.5957 | 0.5 | 0.5794 |
|  | 7 | 0.8 | 0.5957 | 0.3 | 0.5794 |
|  | 8 | 0.9 | 0.5956 | 0.7 | 0.5794 |
|  | 9 | 1.0 | 0.5956 | 0.8 | 0.5794 |
|  | 10 | 0.4 | 0.5956 | 0.9 | 0.5793 |
|  | 11 | 0.5 | 0.5956 | 1.0 | 0.5793 |
| NCCT radiomics  + Radiological signs | 1 | 1.0 | 0.6643 | 1.0 | 0.7769 |
|  | 2 | 0.9 | 0.6640 | 0.6 | 0.7769 |
|  | 3 | 0.8 | 0.6634 | 0.8 | 0.7769 |
|  | 4 | 0.7 | 0.6629 | 0.9 | 0.7769 |
|  | 5 | 0.6 | 0.6627 | 0.3 | 0.7768 |
|  | 6 | 0.5 | 0.6623 | 0.7 | 0.7768 |
|  | 7 | 0.4 | 0.6621 | 0.5 | 0.7768 |
|  | 8 | 0.3 | 0.6608 | 0.4 | 0.7767 |
|  | 9 | 0.2 | 0.6606 | 0.2 | 0.7766 |
|  | 10 | 0.1 | 0.6593 | 0.1 | 0.7756 |
|  | 11 | 0.0 | 0.6531 | 0.0 | 0.7726 |
| Clinical factors | 1 | 0.0 | 0.6234 | 1.0 | 0.7710 |
|  | 2 | 0.1 | 0.6224 | 0.9 | 0.7710 |
|  | 3 | 0.2 | 0.6219 | 0.8 | 0.7710 |
|  | 4 | 0.3 | 0.6218 | 0.7 | 0.7710 |
|  | 5 | 0.5 | 0.6217 | 0.6 | 0.7709 |
|  | 6 | 0.8 | 0.6216 | 0.5 | 0.7709 |
|  | 7 | 0.4 | 0.6216 | 0.4 | 0.7709 |
|  | 8 | 0.9 | 0.6216 | 0.3 | 0.7708 |
|  | 9 | 0.6 | 0.6215 | 0.2 | 0.7706 |
|  | 10 | 0.7 | 0.6214 | 0.1 | 0.7705 |
|  | 11 | 1.0 | 0.6214 | 0.0 | 0.7704 |
| NCCT radiomics  + Clinical factors | 1 | 1.0 | 0.6675 | 0.9 | 0.8098 |
|  | 2 | 0.9 | 0.6673 | 1.0 | 0.8097 |
|  | 3 | 0.8 | 0.6670 | 0.8 | 0.8096 |
|  | 4 | 0.7 | 0.6669 | 0.7 | 0.8095 |
|  | 5 | 0.6 | 0.6666 | 0.6 | 0.8093 |
|  | 6 | 0.5 | 0.6664 | 0.5 | 0.8092 |
|  | 7 | 0.4 | 0.6657 | 0.4 | 0.8090 |
|  | 8 | 0.3 | 0.6651 | 0.3 | 0.8088 |
|  | 9 | 0.1 | 0.6643 | 0.2 | 0.8086 |
|  | 10 | 0.2 | 0.6641 | 0.1 | 0.8079 |
|  | 11 | 0.0 | 0.6560 | 0.0 | 0.8005 |

Table S3: Grid optimisation results for the 11 possible values of the blending hyperparameter α, ranked by their cross-validation AUC (Optimal models ranked at number 1).

| **Criterion** | **Answer** |
| --- | --- |
| Image protocol quality - well-documented image protocols (for example, contrast, slice thickness, energy, etc.) and/or usage of public image protocols allow reproducibility/replicability. | None |
| Multiple segmentations - possible actions are: segmentation by different physicians/algorithms/software, perturbing segmentations by (random) noise, segmentation at different breathing cycles. Analyse feature robustness to segmentation variabilities. | No |
| Phantom study on all scanners - detect inter-scanner differences and vendor-dependent features. Analyse feature robustness to these sources of variability. | No |
| Imaging at multiple time points - collect images of individuals at additional time points. Analyse feature robustness to temporal variabilities (for example, organ movement, organ expansion/shrinkage). | No |
| Feature reduction or adjustment for multiple testing - decreases the risk of overfitting. Overfitting is inevitable if the number of features exceeds the number of samples. Consider feature robustness when selecting features. | Either measure is implemented |
| Multivariable analysis with non radiomics features (for example, EGFR mutation) - is expected to provide a more holistic model. Permits correlating/inferencing between radiomics and non radiomics features. | Yes |
| Detect and discuss biological correlates - demonstration of phenotypic differences (possibly associated with underlying gene–protein expression patterns) deepens understanding of radiomics and biology. | Yes |
| Cut-off analyses - determine risk groups by either the median, a previously published cut-off or report a continuous risk variable. Reduces the risk of reporting overly optimistic results. | No |
| Discrimination statistics - report discrimination statistics (for example, C-statistic, ROC curve, AUC) and their statistical significance (for example, p-values, confidence intervals). One can also apply resampling method (for example, bootstrapping, cross-validation). | A resampling method technique is applied |
| Calibration statistics - report calibration statistics (for example, Calibration-in-the-large/slope, calibration plots) and their statistical significance (for example, P-values, confidence intervals). One can also apply resampling method (for example, bootstrapping, cross-validation). | None |
| Prospective study registered in a trial database - provides the highest level of evidence supporting the clinical validity and usefulness of the radiomics biomarker. | No |
| Validation - the validation is performed without retraining and without adaptation of the cut-off value, provides crucial information with regard to credible clinical performance. | Validation is based on three or more datasets from distinct institutes |
| Comparison to 'gold standard' - assess the extent to which the model agrees with/is superior to the current 'gold standard' method (for example, TNM-staging for survival prediction). This comparison shows the added value of radiomics. | Yes |
| Potential clinical utility - report on the current and potential application of the model in a clinical setting (for example, decision curve analysis). | No |
| Cost-effectiveness analysis - report on the cost-effectiveness of the clinical application (for example, QALYs generated). | No |
| Open science and data - make code and data publicly available. Open science facilitates knowledge transfer and reproducibility of the study. | None |
| **TOTAL SCORE** | 13 (36.11%) |

Table S4: Radiomics Quality Score evaluation of the proposed study.
